# Nitrate, Nitrite, and Ammonium Variability in Drinking Water Distribution Systems

**DOI:** 10.3390/ijerph14030276

**Published:** 2017-03-09

**Authors:** Jörg Schullehner, Leslie Stayner, Birgitte Hansen

**Affiliations:** 1 Geological Survey of Denmark and Greenland (GEUS), C.F. Møllers Allé 8, 8000 Aarhus C, Denmark; bgh@geus.dk; 2 National Centre for Register-Based Research, Department of Economics and Business Economics, School of Business and Social Sciences, Aarhus University, Fuglesangs Allé 4, 8210 Aarhus V, Denmark; 3 School of Public Health, Epidemiology and Biostatistics Division, University of Illinois at Chicago, 1603 W. Taylor Street, Chicago, IL 60612, USA; lstayner@uic.edu

**Keywords:** nitrate, nitrite, ammonium, nitrification, drinking water, exposure assessment, Denmark

## Abstract

Accurate assessments of exposure to nitrate in drinking water is a crucial part of epidemiological studies investigating long-term adverse human health effects. However, since drinking water nitrate measurements are usually collected for regulatory purposes, assumptions on (1) the intra-distribution system variability and (2) short-term (seasonal) concentration variability have to be made. We assess concentration variability in the distribution system of nitrate, nitrite, and ammonium, and seasonal variability in all Danish public waterworks from 2007 to 2016. Nitrate concentrations at the exit of the waterworks are highly correlated with nitrate concentrations within the distribution net or at the consumers’ taps, while nitrite and ammonium concentrations are generally lower within the net compared with the exit of the waterworks due to nitrification. However, nitrification of nitrite and ammonium in the distribution systems only results in a relatively small increase in nitrate concentrations. No seasonal variation for nitrate, nitrite, or ammonium was observed. We conclude that nitrate measurements taken at the exit of the waterworks are suitable to calculate exposures for all consumers connected to that waterworks and that sampling frequencies in the national monitoring programme are sufficient to describe temporal variations in longitudinal studies.

## 1. Introduction

Nitrate and nitrite in drinking water can have negative human health impacts, while ammonium is not of direct importance for human health [1]. The current drinking water standards in Denmark follow the European Union and World Health Organization’s guidelines. Public waterworks are accountable for the drinking water quality in the distribution net until a pipe reaches private property (in practice, the water meter of the customer). For nitrate, the drinking water standard is 50 mg/L at exit waterworks, entry to private property, and at the consumers’ taps. For nitrite, the drinking water standard is 0.01 mg/L at exit waterworks and 0.1 mg/L at entry to private property and at the consumers’ taps. In Denmark, up to 0.1 mg/L nitrite measured at the exit of the waterworks are permissible, if it can be documented that the drinking water standard at the entry to private property and the consumers’ taps is complied with [2]. The Danish drinking water standard for ammonium is 0.05 mg/L. Ammonium concentrations up to 0.5 mg/L are accepted, if there is no filtration step at the waterworks and ammonium is not converted to nitrite in the distribution system [2].

Nitrate and nitrite drinking water standards are set to protect infants from the acute condition methemoglobinemia, based on clinical evidence [1]. However, due to the role of nitrate and nitrite as precursors of genotoxic *N*-nitroso compounds in endogenous nitrosation, nitrate and nitrite in drinking water are suspected to cause cancer in the gastrointestinal and urinary tract and at other sites [3,4]. The International Agency for Research on Cancer classified nitrate and nitrite as probably carcinogenic to humans (group 2A) under conditions that result in endogenous nitrosation [5]. While there is a plausible etiological explanation with endogenous nitrosation, epidemiological evidence from studies on humans has been inconclusive [3,4]. However, two recent studies show an association between nitrate in drinking water and cancer, in particular colorectal cancer [6], and bladder cancer [7]. There is also evidence that nitrate in drinking water may be associated with an increased risk of birth defects [8] and other adverse reproductive outcomes [9].

Nitrate is a common pollutant in Danish ground- and drinking water, originating from agricultural activities. Historically, concentrations have been increasing in ground- and drinking water on a national-level until the 1980s, after which concentrations have been decreasing, owing to a more efficient use of nitrogen fertilizers and stronger environmental regulations [10]. In oxic groundwater and oxygenated water systems, nitrate (NO_3_^−^) is the stable form of nitrogen, while nitrite (NO_2_^−^) is relatively unstable [11]. Nitrite and ammonium concentrations are comparatively low and indicate chemical instability, smaller loadings, and/or limited mobility, as noted by Nolan and Stoner (2000) [12]. Nitrite in groundwater originates from incomplete reduction of nitrate in the anoxic nitrate reducing parts of aquifers, while ammonium originates from geogenic degradation of organic matter in the reduced part of aquifers [13]. However, elevated concentrations of ammonium in groundwater samples may indicate faecal pollution [1].

**Figure 1:**
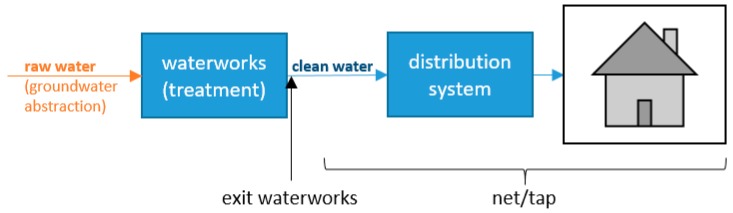
Schematic overview of sampling locations.

Danish drinking water production is based exclusively on groundwater, most often only with simple treatment (aeration and sand filtration), and no chlorination. At the waterworks, ammonium is removed from the groundwater (raw water, see Figure 1), most often by biological nitrification: Equation (1) shows the first step of nitrification of ammonium to nitrite and Equation (2) the second step to nitrate.

2NH_4_^+^ + 3O_2_ → 2NO_2_^−^ + 2H_2_O + 4H^+^, (1)

2NO_2_^−^ + O_2_ → 2NO_3_^−^ (2)

Nitrification may also occur in the distribution system. In the following, we will use the term ammonium for the sum of both ammonia (NH_3_) and ammonium (NH_4_^+^), since ammonium is the predominant form at typical drinking water pH and temperature.

Assessing the historical exposure of study participants naturally holds challenges, as scientists need to rely on historical databases that were not originally designed for the use in epidemiological studies, but rather for regulatory purposes. In Denmark, the publicly-accessible national geodatabase Jupiter (www.geus.dk) provides a valuable source of historical drinking water quality data. It was shown that this database can be used to calculate nitrate exposures of all Danish individuals decades back in time [14]. However, drinking water quality is not continuously monitored. Data coverage depends on the drinking water production volume of the public drinking water supplies: while the smallest waterworks producing less than 10,000 m^3^ per year only need to take drinking water quality samples every second year, larger waterworks are required to take between one sample per year and up to several samples per month [2,15]. The sampling frequency may therefore not detect seasonal variations in concentrations and could, if seasonal variation exists, potentially introduce errors in the exposure assessment and thereby reduce the quality of epidemiological studies. Studies on seasonal variation of nitrate and nitrite in drinking water are few. A study conducted in four Midwestern United States found seasonal patterns in nitrate-nitrite concentrations in drinking water, with the highest concentrations observed in winter and early spring [9]. Stayner and colleagues [9] also point to the possibility of water supply managers deliberately sampling at times of especially high or low concentrations, reducing the representativeness of the data. Another study of 853 wells in the Midwestern United States concluded that a single measurement of nitrate was representative for longer periods of exposure [16]. A study of nitrate levels in twelve water-supply wells in Yucatan, Mexico found a seasonal pattern that the authors contributed to dilution effects during the pronounced wet season [17].

Another challenge is the sampling location. Drinking water samples are taken at the exit of the waterworks (after all treatment steps), and to some extent within the distribution system and at the consumers’ taps. Often, epidemiologists assume that nitrate behaves conservatively after leaving the waterworks, i.e., information on nitrate concentrations measured at the exit of the waterworks, within the system, and at the consumers’ taps are equally appropriate to describe the nitrate concentration in the whole supply system. Studies on this issue are lacking, a recent Dutch study presented at the 3rd International Symposium on Environment and Health in 2016 supported the assumption of a very high correlation between nitrate concentrations at the waterworks and the taps [18].

Assumptions made in epidemiologic studies of negligible intra-distribution system variability and the lack of seasonal variability may be used to challenge the exposure assessments of these studies. In this article, we assess the validity of these assumptions.

## 2. Materials and Methods

We retrieved drinking water quality measurements for all Danish public waterworks for nitrate, nitrite, and ammonium/ammonia from the publicly accessible national geodatabase Jupiter on 20 December 2016. Samples were taken between 1 January 2007 and 7 December 2016. Samples were coded on their sampling location, either at exit waterworks, or within the distribution system or at the consumers’ taps. Samples were analysed by certified laboratories using standard methods before being uploaded to Jupiter.

**Figure 2:**
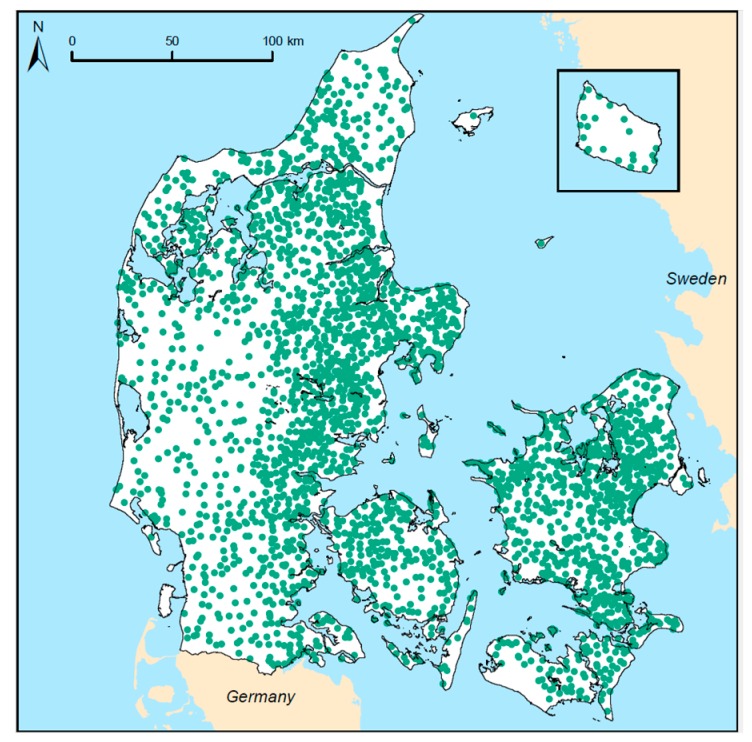
Locations of the 2948 public waterworks included in this study.

Table 1 summarizes the resulting dataset. Waterworks have a well-defined sampling tap just before the produced drinking water is pumped into the distribution net, here called “exit waterworks”. The majority of samples were taken at this tap at the exit of the waterworks. Samples taken within the distribution net (“net”) and at the consumers’ taps (“tap”) were combined for further analyses. Figure 1 shows the locations schematic. The final dataset included nitrate, nitrite, and ammonium measurements from 2948 individual public waterworks, covering all of Denmark, see Figure 2.

**Figure 3:**
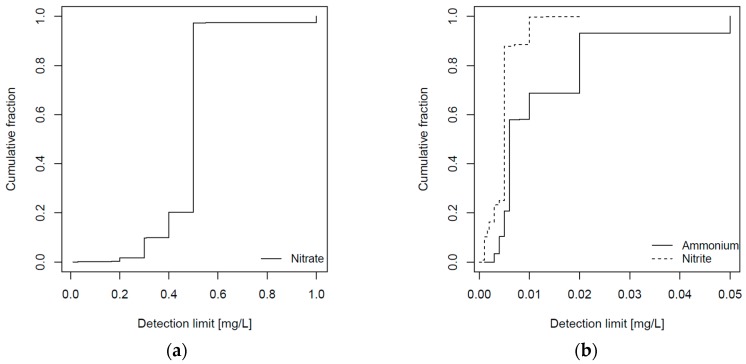
Cumulative fraction of detection limits of samples below the detection limit for (**a**) nitrate (*N* = 9144) and (**b**) nitrite (*N* = 35,690) and ammonium (*N* = 20,583).

Figure 3 shows the cumulative fraction of the varying detection limits for (a) nitrate and (b) nitrite and ammonium. We imputed half the value of the detection limit for measurements below the detection limit. As shown in Table 1, a substantial fraction of samples were below the detection limit, in particular for nitrite (65%) and ammonium (44%). We therefore conducted sensitivity analyses by excluding all measurements below the detection limit.

To assess the variability within drinking water supply systems, we matched samples taken within the distribution net or at the consumers’ taps with the closest taken sample at the exit of the respective waterworks. We only included matched sample-pairs, if the samples were taken within a maximum time difference of 30 days.

Outliers were analysed and removed, when it could be established that large differences between concentrations at exit waterworks and within the system were due to connection/closure of boreholes (nitrate: four outliers, nitrite: seven outliers, ammonium: three outliers). We used linear regression to assess the association between concentrations at exit waterworks and within the distribution network or at the consumers’ taps. To assess the seasonal variability, we created box and whisker plots of the respective concentrations by calendar month. We used IPython [19] to retrieve data from Jupiter, R 3.3 [20] for data management and statistical computations, and R 3.3 and matplotlib [21] for creation of graphs.

## 3. Results

### 3.1. Variability within the Distribution System

**Figure 4:**
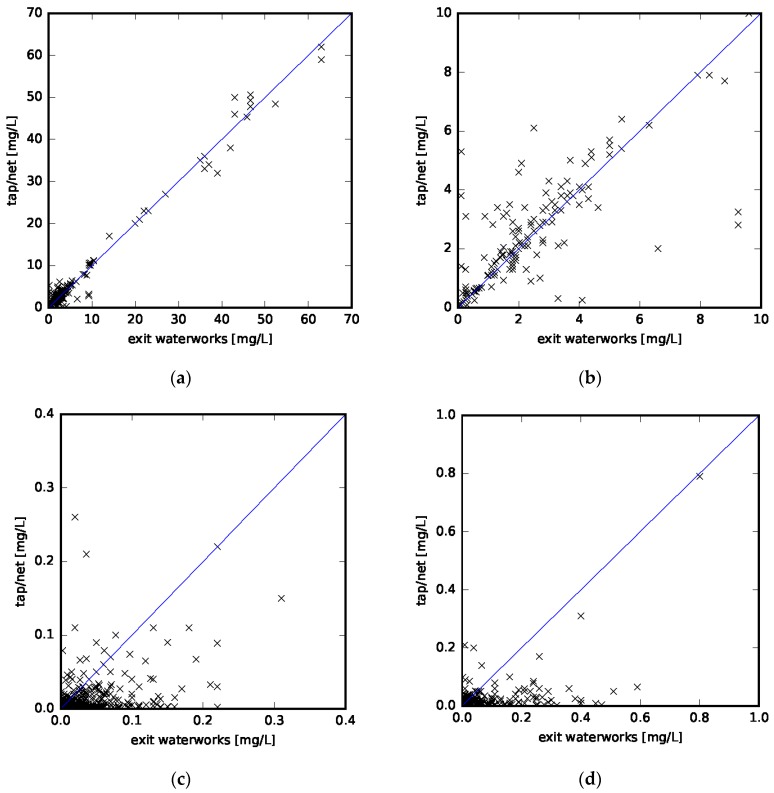
Concentrations at the waterworks versus concentrations in the distribution system or at the consumers’ taps for (**a**) Nitrate (*N* = 195); (**b**) Nitrate zoomed below 10 mg/L; (**c**) Nitrite (*N* = 1562); (**d**) Ammonium (*N* = 391). The blue line indicates the identity line.

Table 2 shows the results of the linear regression analyses for nitrate, nitrite, and ammonium. For nitrate, only 195 sample-pairs matched the criterion of being measured at the waterworks and within the distribution net/tap within one month. Significantly more sample-pairs were available for nitrite, probably due to the obligation of waterworks to monitor whether nitrite concentrations comply with the drinking water standards within the system and at the taps. In Figure 4, concentrations of nitrate, nitrite, and ammonium measured at exit waterworks are plotted against measurements taken within the distribution net or at the consumers’ taps. The blue line indicates the identity line: water supply systems below this line had higher concentrations measured at exit waterworks than within the distribution net or at the taps, while water supply systems above the line had higher concentrations measured within the distribution net or at the taps than at exit waterworks.

### 3.2. Seasonal Variability

**Figure 5:**
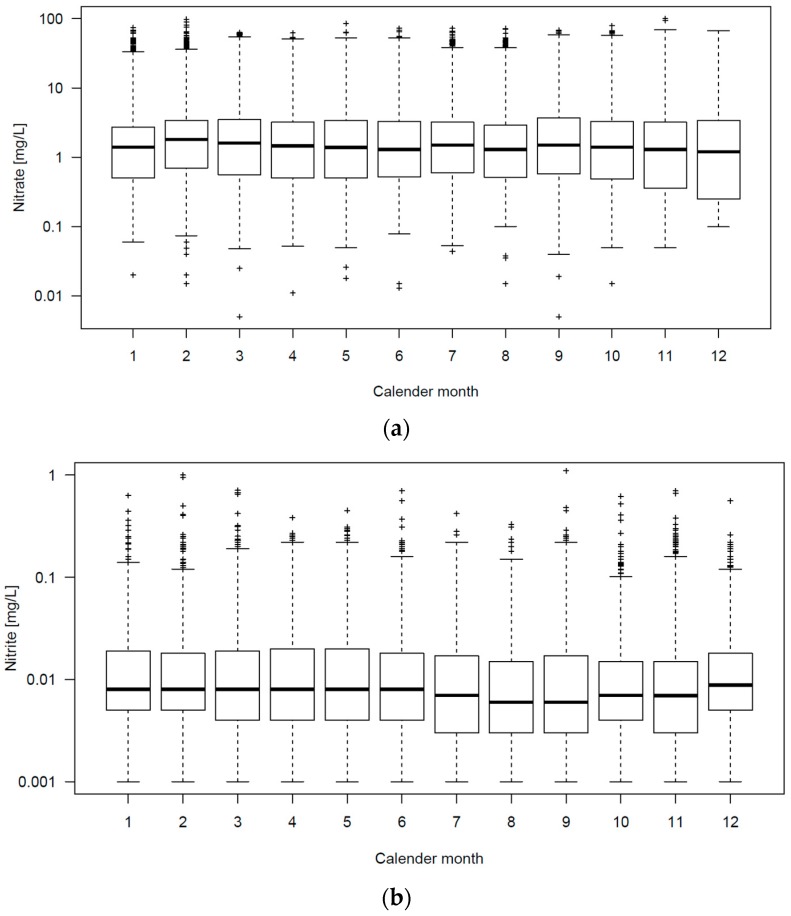

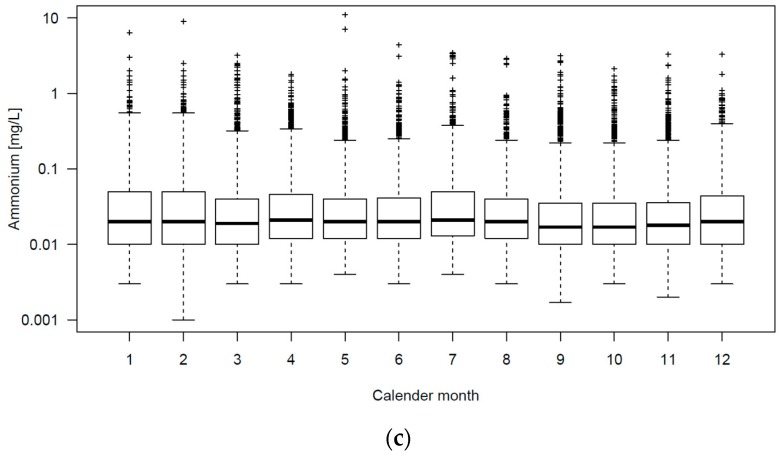
Box and whisker plots of: (**a**) Nitrate (*N* = 42,823); (**b**) Nitrite (*N* = 54,859); (**c**) Ammonium (*N* = 47,052) concentrations in public supplies by calendar month.

Figure 5 shows box and whisker plots nitrate, nitrite, and ammonium concentrations by calendar month.

## 4. Discussion

Nitrate concentrations at exit waterworks and within the distribution net/taps are highly correlated and follow a nearly 1:1 association, as can be seen in Figure 4a and Table 2. Sensitivity analyses excluding measurements below the detection limit showed similar results (not shown here). Therefore, the assumption of using either sampling location to estimate nitrate concentrations within the whole system holds. This allows us to take advantage of the significant higher number of samples taken at exit waterworks. Nitrate levels do neither increase nor decrease in the distribution system. These findings live up to our expectations. Danish waterworks do not use chloramination and the observed ammonium levels are comparatively low, which prevents significant nitrate formation in biofilms as described by Barry and colleagues [22]. Denitrification is not expected, as drinking water generally lives up to regulation demands for oxygen levels of at least 5 mg/L within the whole distribution system [2].

Both nitrite and ammonium levels are lower within the distribution net and tap than at exit waterworks. The generally low nitrite and ammonium concentrations compared with nitrate concentrations are noteworthy. While 21% of nitrate measurements were below the detection limit, this was the case in 65% of nitrite and 44% of ammonium measurements. Again, this is due to the Danish drinking water structure, based exclusively on groundwater, in which nitrite and ammonium concentrations are low compared with surface water sources [23]. The general trend of lower nitrite and ammonium concentrations in the distribution system and tap compared with exit waterworks can be explained by nitrification as shown in Equations (1) and (2). This will lead to an increase in nitrate levels. However, since nitrite and ammonium levels are comparatively low, the increase in nitrate levels is not detected here, since the predominant source of nitrate originates from groundwater and not nitrification in the distribution system. As an example, complete nitrification of the highest detected levels of nitrite would increase nitrate concentrations by around 0.4 mg/L. Complete nitrification of the highest detected levels of ammonium would increase nitrate concentrations by less than 3 mg/L. Compared with the overall nitrate concentration range of more than 60 mg/L, nitrification in the distribution net will therefore only be a small contribution. However, at lower nitrate levels as shown in Figure 4b, this contribution may lead to a noticeable increase in nitrate concentrations.

We do not observe seasonal variability in concentrations for nitrate, nitrite, or ammonium, as shown in Figure 5. This is in contrast to two earlier studies, as mentioned above [9,17]. We explain the results partly by the exclusive use of groundwater, with the majority being between 10 and 60 years old and the more attenuated precipitation patterns as compared with the Mexican study that found seasonal variability for nitrate concentrations [17]. However, as earlier studies showed, long-term trends in nitrate concentrations in Danish ground- and drinking water do exist [14,24], which is why a single nitrate measurement to estimate long-term exposure is not deemed suitable. For the purposes of epidemiological studies, the Danish national drinking water regulation provides an appropriate sampling frequency, with at least one sample every second year at the smaller public waterworks and multiple samples per year for the larger waterworks. In combination with the high quality data on residence of all Danish residents, with full address information from 1977 and onwards [25,26], the data on drinking water quality in the national geodatabase Jupiter provides a valuable source for exposure estimation in studies on long-term health effects of drinking water quality.

## 5. Conclusions

Drinking water nitrate levels measured from samples taken at the exit of the waterworks, within the distribution net, or at the consumers’ taps are representative of the nitrate levels within the whole system. The typically much higher number of samples taken at the waterworks for monitoring purposes can be used to estimate concentrations at the consumers’ taps. Nitrate concentrations do not vary seasonally, thus also single nitrate samples represent longer periods. Therefore, regulatory databases, such as the Danish national geodatase Jupiter, are suitable for nitrate exposure estimations in epidemiological studies; particularly in settings similar to the Danish drinking water infrastructure fully depending on groundwater without disinfection.

Nitrite and ammonium exposures are less often estimated in epidemiological studies. In Danish drinking water, levels are low. Generally, concentrations of both nitrite and ammonium are lower in the distribution net than at the exit of the waterworks. We attribute this to nitrification processes in the distribution net. However, the resulting changes in nitrate levels are not notably influenced by this nitrification, since the main source of nitrate in drinking water is groundwater and not in-system redox processes.

## Figures and Tables

**Table 1:** Nitrate, nitrite, and ammonium measurements taken in public water supply systems between 2007 and 2016 by sampling location and percentage of samples below detection limit (<LOD).

Compound	Exit Waterworks	Net	Tap	Total	<LOD (%)
Nitrate	41,638	1030	155	42,823	21
Nitrite	43,763	9866	1230	54,859	65
Ammonium	43,454	3443	155	47,052	44

**Table 2:** Linear regression coefficients and 95% confidence intervals of nitrate and nitrite concentrations at exit waterworks versus within distribution system and consumers’ taps.

Compound	Slope	Intercept	*R*^2^	*N*
Nitrate	0.98 [0.96;1.00]	0.24 [−0.01;0.49]	0.98	195
Nitrite	0.22 [0.20;0.24]	0.00 [0.00;0.00]	0.19	1562
Ammonium	0.23 [0.18;0.27]	0.01 [0.00;0.01]	0.22	391

## References

[1] World Health Organization (2011). Guidelines for Drinking-Water Quality.

[2] Ministry of Environment and Food of Denmark Ministerial Order on Water Quality and Monitoring of Water Supply Plants. https://www.retsinformation.dk/Forms/R0710.aspx?id=180348.

[3] Ward M.H., de Kok T.M., Levallois P., Brender J., Gulis G., Nolan B.T. (2005). Workgroup Report: Drinking-Water Nitrate and Health—Recent Findings and Research Needs. Environ. Health Perspect..

[4] Villanueva C.M., Kogevinas M., Cordier S., Templeton M.R., Vermeulen R., Nuckols J.R., Nieuwenhuijsen M.J., Levallois P. (2014). Review Assessing Exposure and Health Consequences of Chemicals in Drinking Water: Current State of Knowledge and Research Needs. Environ. Health Perspect..

[5] International Agency for Research on Cancer (2010). IARC Monographs on the Evaluation of Carcinogenic Risks to Humans. Volume 94—Ingested Nitrate and Nitrite, and Cyanobacterial Peptide Toxins.

[6] Espejo-Herrera N., Gràcia-Lavedan E., Boldo E., Aragonés N., Pérez-Gómez B., Pollán M., Molina A.J., Fernández T., Martín V., La Vecchia C. (2016). Colorectal cancer risk and nitrate exposure through drinking water and diet. Int. J. Cancer.

[7] Jones R.R., Weyer P.J., DellaValle C.T., Inoue-Choi M., Anderson K.E., Cantor K.P., Krasner S., Robien K., Beane Freeman L.E., Silverman D.T. (2016). Nitrate from drinking water and diet and bladder cancer among postmenopausal women in Iowa. Environ. Health Perspect..

[8] Brender J.D., Olive J.M., Felkner M., Suarez L., Marckwardt W., Hendricks K.A. (2004). Dietary nitrites and nitrates, nitrosatable drugs, and neural tube defects. Epidemiology.

[9] Stayner L.T., Almberg K., Jones R., Graber J., Pedersen M., Turyk M. (2017). Atrazine and nitrate in drinking water and the risk of preterm delivery and low birth weight in four Midwestern states. Environ. Res..

[10] Hansen B., Dalgaard T., Thorling L., Sørensen B., Erlandsen M. (2012). Regional analysis of groundwater nitrate concentrations and trends in Denmark in regard to agricultural influence. Biogeosciences.

[11] World Health Organization (2011). Nitrate and Nitrite in Drinking-Water. Background Document for Development of WHO Guidelines for Drinking-Water Quality.

[12] Nolan B.T., Stoner J.D. (2000). Nutrients in Groundwaters of the Conterminous United States, 1992–1995. Environ. Sci. Technol..

[13] Appelo C.A.J., Postma D. (2005). Geochemistry, Groundwater and Pollution.

[14] Schullehner J., Hansen B. (2014). Nitrate exposure from drinking water in Denmark over the last 35 years. Environ. Res. Lett..

[15] Danish Nature Agency (2014). Kvaliteten af det Danske Drikkevand for Perioden 2011–2013 (Quality of the Danish Drinking Water for the Period 2011–2013, in Danish).

[16] Ruckart P.Z., Henderson A.K., Black M.L., Flanders W.D. (2008). Are nitrate levels in groundwater stable over time?. J. Expo. Sci. Environ. Epidemiol..

[17] Pachero J., Marín L., Cabrera A., Steinich B., Escolero O. (2001). Nitrate temporal and spatial patters in 12 water-supply wells, Yucatan, Mexico. Environ. Geol..

[18] Van der Aa M. Big data epidemiology: Drinking water quality in relation to health statistics in the Netherlands. Proceedings of the 3rd International Symposium on Environment and Health.

[19] Pérez F., Granger B.E. (2007). IPython: A System for Interactive Scientific Computing. Comput. Sci. Eng..

[20] R Core Team (2016). R: A Language and Environment for Statistical Computing.

[21] Hunter J.D. (2007). Matplotlib: A 2D graphics environment. Comput. Sci. Eng..

[22] Berry D., Xi C., Raskin L. (2006). Microbial ecology of drinking water distribution systems. Curr. Opin. Biotechnol..

[23] Bower E.J., Crowe P.B. (1988). Biological Processes in Drinking Water Treatment. J. AWWA.

[24] Hansen B., Thorling L., Dalgaard T., Erlandsen M. (2011). Trend Reversal of Nitrate in Danish Groundwater—A Reflection of Agricultural Practices and Nitrogen Surpluses since 1950. Environ. Sci. Technol..

[25] Pedersen C.B., Gøtzsche H., Møller J.Ø., Mortensen P.B. (2006). The Danish Civil Registration System—A cohort of eight million persons. Dan. Med. Bull..

[26] Pedersen C.B. (2011). The Danish Civil Registration System. Scand. J. Public Health.

